# Improvement in Clinical Symptoms and Fecal Microbiome After Fecal Microbiota Transplantation in a Dog with Inflammatory Bowel Disease

**DOI:** 10.2147/VMRR.S230862

**Published:** 2019-12-02

**Authors:** Ayaka Niina, Ryoko Kibe, Ryohei Suzuki, Yunosuke Yuchi, Takahiro Teshima, Hirotaka Matsumoto, Yasushi Kataoka, Hidekazu Koyama

**Affiliations:** 1 Laboratory of Veterinary Internal Medicine, School of Veterinary Medicine, Faculty of Veterinary Medicine, Nippon Veterinary and Life Science University, Musashino, Tokyo, Japan; 2 Laboratory of Veterinary Microbiology, School of Veterinary Medicine, Faculty of Veterinary Medicine, Nippon Veterinary and Life Science University, Musashino, Tokyo, Japan

**Keywords:** fecal microbiota transplantation, inflammatory bowel disease, canine, dysbiosis, microbiome, diversity

## Abstract

**Purpose:**

Recently, fecal microbiota transplantation (FMT) has been tested in veterinary medicine as a treatment option for multiple gastrointestinal (GI) diseases, such as inflammatory bowel disease (IBD). However, there are no reports of changes in the microbial diversity of fecal microbiome after treatment with FMT in canine IBD cases. Moreover, little is known about the long-term efficacy and safety of FMT treatment for dogs. Herein, we present a case of canine intractable IBD treated with repeated, long-term FMT.

**Patients and methods:**

The patient was a 10-year-old, neutered, male, 4-kg Toy Poodle with a prolonged history of vomiting and diarrhea. Fecal examination for pathogens was negative. Despite treatment with multiple antibacterial and antidiarrheal agents, the patient showed no improvement. Endoscopic mucus sampling diagnosed a case of lymphocytic-plasmacytic duodenitis, ie, idiopathic IBD. Eventually, we performed periodic, long-term fecal microbiota transplantation of fresh donor feces collected from a 4-year-old, 32.8-kg, neutered male Golden Retriever by rectal enema. Additionally, we performed 16S rRNA sequence analysis, before and after FMT, to evaluate the microbiome diversity.

**Results:**

Fecal microbiome diversity after FMT resembled that of the healthy donor dog’s fecal microbiome, before FMT, which led us to conclude that the fecal microbiome in our patient normalized with FMT. Moreover, the clinical symptoms improved remarkably with regard to the changes in the fecal microbiome. Additionally, we noted no observable side effects during FMT treatment.

**Conclusion:**

This report indicates the efficacy and safety of long-term, periodic FMT for a case of canine IBD based on attenuation of clinical symptoms and changes in fecal microbiome diversity. Therefore, FMT could be chosen as a treatment option for IBD in canines in the future.

## Introduction

Inflammatory bowel disease (IBD) is a common cause of idiopathic, chronic, and relapsing gastrointestinal (GI) disease in dogs.^1^ The most common histological change associated with IBD is lymphoplasmacytic inflammation; however, eosinophilic and neutrophilic inflammation can also be identified.^2^ The cause of IBD is unknown, but it is believed to be secondary to the complex interplay between genetics, immune dysregulation, and environmental factors, including the GI microbiome.^3^ In human medicine, fecal microbiota transplantation (FMT) has been reported as an effective treatment for recurrent *Clostridium difficile* infections.^4^

In FMT, fecal matter is collected from a tested healthy donor, mixed with saline or other solutions, strained, and administered to a patient by colonoscopy, endoscopy, sigmoidoscopy, or enema.^5^–^7^ In veterinary medicine, FMT has recently been tested as a treatment option for multiple GI diseases, such as IBD.^8^ However, changes in microbiome diversity after treatment with FMT have not been reported for canine IBD. Moreover, whether FMT is an effective and safe treatment for canine IBD still remains unknown.

Herein, we present a case of canine intractable IBD treated with repeated long-term FMT. Additionally, we performed 16S rRNA sequence analysis to evaluate the microbiome diversity before and after treatment with FMT.

## Case Description

### Patient Characteristics

A 10-year-old, neutered, male, 4.0-kg Toy Poodle presented with a prolonged history of vomiting and diarrhea. Real-time PCR analysis (IDEXX Laboratories, Inc., Tokyo, Japan) of the dog’s fecal sample was negative for *Cryptosporidium* spp., *Giardia* spp., *Clostridium perfringens* α toxin, *Clostridium difficile* toxins A and B, *Campylobacter jejuni, Campylobacter coli, Salmonella* spp., canine parvovirus type 2, canine distemper virus, and canine enteric coronavirus genes.

### Initial Treatment

The dog was treated with metronidazole (14.8 mg/kg PO, q12 h) and an antidiarrheal compound for 7 days; however, the consistency of the stool did not improve. Subsequently, the antibiotic treatment regimen was changed to orbifloxacin (5 mg/kg PO, q24 hr) and an antiflatulent, administered for 14 days. However, the diarrhea persisted, and his condition did not improve. Eventually, the patient was referred to Nippon Veterinary and Life Science University for an endoscopy and further treatment. Thus, we underwent the canine upper endoscopy and obtained the gastric and duodenal mucosa biopsies. Tylosin (10 mg/kg, PO, q12 hr) was administered for 14 days, before pathological examinations; however, the stool consistency did not improve. Endoscopic mucosal sampling revealed lymphocytic-plasmacytic duodenitis, and idiopathic IBD was diagnosed. We treated the patient with prednisolone/cyclosporin (2 mg/kg, PO, q24 hr/10 mg/kg, PO, q24 hr) for 9 months. Stool consistency initially showed improvement; however, the patient’s clinical condition did not stabilize without the medicine, and FMT was prescribed. All pharmacotherapeutic treatments were discontinued 1 week before we performed FMT, which kept discontinued throughout the trial.

### Donor Dog Characteristics

We collected fresh donor feces from a 4-year-old, 32.8-kg, neutered, male Golden Retriever. The donor dog was determined to be in good health after undergoing physical and clinical examinations, complete blood count, serum biochemical analysis, radiography, abdominal ultrasound, and fecal examination. The donor dog’s feces were also evaluated with real-time PCR analysis (IDEXX Laboratories, Inc.), and the analysis revealed no pathogenic microbes.

### FMT Protocol

We collected the donor’s feces within 6 hrs before performing FMT. Immediately after fecal collection, approximately 12 g (3 g/kg) of feces was dissolved in 36 mL (9 mL/kg) of Ringer’s solution. The slurry was then passed through a sterilized gauze to filter out particulate matter. Totally, we administered 40 mL (10 mL/kg) of the slurry to our patient during each FMT procedure. Generally, FMT was performed either orally (eg, nasoduodenal intubation and enteroscopy) or rectally (ie, rectal enema and colonoscopy).^9^ Additionally, it has been reported that colonoscopy for FMT has excellent cure rates and can be well tolerated.^10^ Therefore, we chose rectal enema as the route of administration for this case due to efficacy and safety.

There are no reports on the optimum dose and treatment interval for FMT procedures; thus, we determined the interval of each FMT by observing the symptoms and improvement in stool consistency in this case. The first FMT was performed on day 0. Totally, we treated the dog with FMT 9 times within 6 months. As a result of the 6-month trial, stool consistency has shown improvement and stabilized by FMT for up to 63 days. However, after 25 days from FMT, the patient’s owner recognized the difference in fecal smell and frequency of the flatus. From the observations, we decided to perform FMT once every 3 weeks.

### Clinical Symptoms

**Figure 1:**
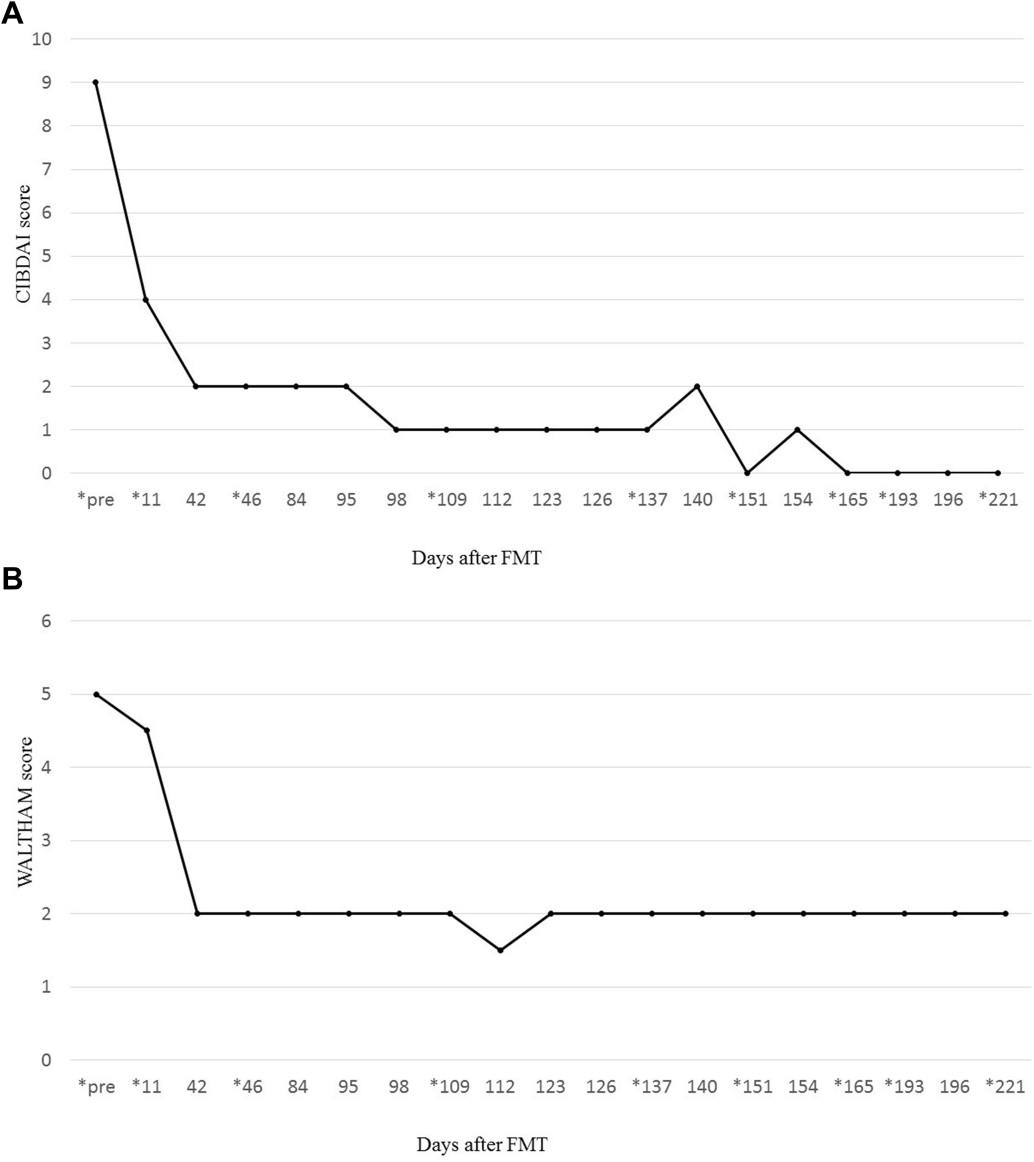
(**A**) Clinical observations according to the canine inflammatory bowel disease activity index score. A score of 3 or less was considered normal. * indicates the date of each fecal microbiota transplantation (FMT) procedure conducted. Of note, the patient with inflammatory bowel disease (IBD) achieved a normal score from day 42 of FMT throughout the remainder of the observation period. (**B**) Clinical observations according to the Waltham^TM^ Feces Scoring System. A score of 3.5 or less was considered normal. *indicates the date of each FMT procedure conducted. Of note, the patient with IBD achieved a normal score from day 42 throughout the remainder of the observation period.

We evaluated the changes in clinical symptoms using the canine inflammatory bowel disease activity index (CIBDAI) and Waltham^TM^ fecal conditioning score. From days 3 to 11 post-FMT, the patient’s frequency of vomiting and defecation decreased, and stool showed normal consistency. The CIDBAI score improved from 9 to 4, while the Waltham^TM^ score improved from 5 to 2 (Figure 1A and B). No adverse effects occurred throughout the course of treatment with FMT.

### Fecal Microbiome Analysis

**Figure 2:**
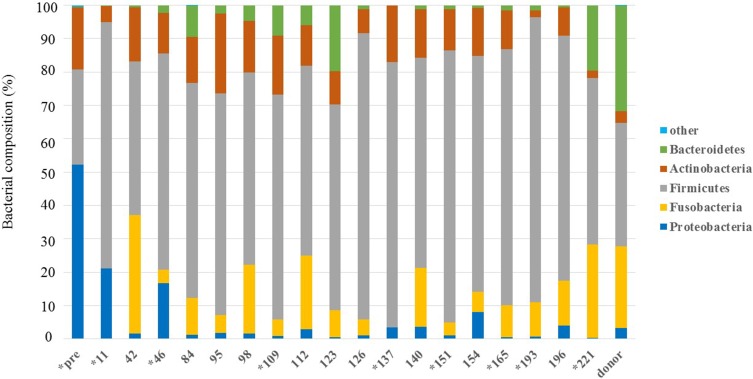
Rarefaction analysis of V3-V4 16S rRNA gene sequences. Rarefaction analysis using a fecal sample from the dog with inflammatory bowel disease (IBD) showed changes in bacterial populations at, before, and after fecal microbiota transplantation (FMT) compared to the healthy donor dog. * indicates the date of each FMT procedure conducted.

Rarefaction analysis of 16S rRNA gene sequences was performed using MiSeq Reporter (software 2.6.2.3 Illumina, Inc.) to assess microbiome diversity. Raw sequence data were screened, trimmed, and filtered with default settings using the QIIME 2 view tool. The analysis was performed on a randomly selected subset of 30,213 ± 4721 sequences from each sample. The V3-V4 16S rRNA gene sequences were analyzed for the identification of select bacterial groups, including Actinobacteria, Bacteroidetes, Firmicutes, Fusobacteria, Proteobacteria, and other bacteria. The analysis showed a difference in the proportion of bacterial populations in the feces before FMT, compared to that after FMT and with the donor fecal sample (Figure 2). Before FMT, the predominant bacterial phylum was Proteobacteria (52.2%) and Firmicutes, comprising 28.4% of the total bacterial population. Actinobacteria comprised 18.6%, Bacteroidetes comprised 0.3%, while Fusobacteria was not detected. On day 42 after FMT, the proportion of Proteobacteria decreased to 1.5%, whereas Fusobacteria significantly increased to 35%. Moreover, the proportion of the other dominant bacterial groups, Firmicutes and Bacteroidetes, increased. The microbiome of the feces from the patient after completion of FMT treatment was similar to that of the healthy donor dog. Additionally, these changes in microbial diversity coincided with the improvement of CIBDAI scores.

## Discussion

The patient dog described in this case report presented with a long history of vomiting and diarrhea. The upper endoscopic examination revealed lymphocytic-plasmacytic duodenitis, ie, idiopathic IBD. Conventional treatment, consisting of an antiflatulent agent, an antidiarrheal agent, antibiotics, and anti-inflammatory drugs, did not improve the dog’s symptoms of diarrhea and vomiting. The use of FMT was employed and the symptoms improved after FMT treatment. Totally, we treated the dog with FMT 9 times within 6 months. From observations, we decided to perform FMT once every 3 weeks. Herein, we concluded that a dog with intractable IBD achieved clinical resolution with repeated, long-term FMT.

We performed 16S rRNA sequence analysis to evaluate the microbial diversity in the feces of the dog with IBD after each round of treatment with FMT. A previous study reported that the proportion of Proteobacteria was 5–7% and Fusobacteria was 23–40% in the fecal microbiome of healthy dogs.^11^ In our study, the proportion of Proteobacteria was 3.2% and that of Fusobacteria was 24.5% in the feces of the healthy donor dog. Before FMT, the proportion of Proteobacteria was high (52.2%) in the feces of our patient; however, Fusobacteria was undetectable. We identified a significant difference in the fecal microbiome diversity between our patient and the healthy dog. Dysbiosis refers to the abnormal constitution of the microbiome, and is commonly observed in animals with acute or chronic gastrointestinal diseases.^12^–^14^ Furthermore, it is generally assumed that *Gammaproteobacteria*, included in Proteobacteria, evoke dysbiosis and cause an imbalance of the enteral environment.^8^,^15^,^16^ There was a lower proportion of Fusobacteria in the patient’s feces than in that of the healthy dog.^8^,^11^,^16^ However, several studies have reported that Fusobacterium has been implicated as a proinflammatory pathogen^17^–^19^ and has been found in a higher abundance of IBD patients in mouse model and human.^19^ It was suggested that Fusobacterium caused colitis-associated colorectal cancer in the mouse model and the human. Although Fusobacteria may be the risk factor in mouse and human, the low rate of Fusobacteria may be specific for canine IBD. Therefore, the population of Proteobacteria and Fusobacteria may be related to the clinical GI symptoms in the present case of canine IBD. We propose that a high proportion of Proteobacteria and a low proportion of Fusobacteria may be a characteristic feature of canine IBD.

After the FMT procedure, there was a significant decrease in the proportion of Proteobacteria and a significant increase in the proportion of Fusobacteria and other dominant bacterial groups (Firmicutes and Bacteroidetes). This fecal microbiome diversity was similar to that of the donor’s fecal microbiome, which allows us to conclude that the diversity of the fecal microbiome was normalized by FMT in the present case of canine IBD. Moreover, our observations confirmed that changes in the fecal microbiome diversity indeed affected the clinical symptoms of IBD, and hence, contributed to the efficacy of FMT treatment. Although reports indicate FMT to be a safe procedure for short-term treatment,^2^ only few studies have reported on the safety of long-term FMT procedures. Since the completion of the 10th round, we have continued performing FMT regularly, once every 3 weeks, till date. There have been no observed side effects, including diarrhea, vomiting, or abdominal pain, associated with the FMT procedure. Therefore, we report that long-term FMT treatment for canine IBD is safe.

In this study, we used feces from only one healthy donor dog for FMT. Future studies should examine the efficacy and safety of FMT using feces from multiple healthy donors. In addition, we did not perform an endoscopy on our patient after FMT due to lack of consent from the owner. Therefore, we were unable to confirm any changes in the intestinal mucosa resulting from FMT in this case. FMT is trial treatment in this case, hence it does not cost. Also, there is no risk of anesthesia and no side effect is observed up to this point of this trial. Therefore, we will continue performing FMT every 3 weeks, as far as the improvement of the symptom keeps.

In conclusion, we show the efficacy and safety of FMT in this case report. We conclude that FMT should be considered as a treatment option for other canine IBD or intractable IBD cases in the future.

## Abbreviations

CIBDAI, canine inflammatory bowel disease activity index; FMT, fecal microbiota transplantation; IBD, inflammatory bowel disease; GI, gastrointestinal.
